# Does the British Columbia Farmers’ Market Nutrition Coupon Program improve the diet quality and food security of parents and children with lower incomes? Protocol for a dose-response, pragmatic randomized controlled trial

**DOI:** 10.1177/02601060261473062

**Published:** 2026-09-12

**Authors:** Dana Lee Olstad, David JT Campbell, Tolulope T Sajobi, Heather O’Hara, Peter Leblanc, Gavin R McCormack, Leticia Radin-Pereira, Yun Yun Lee, Michelle L Aktary, Leia M Minaker, Eldon Spackman

**Affiliations:** 1 Department of Community Health Sciences, 70401 Cumming School of Medicine, University of Calgary, Calgary, AB, Canada; 2 Department of Medicine, Cumming School of Medicine, University of Calgary Foothills Medical Centre—North Tower, Calgary, AB, Canada; 3 Department of Cardiac Sciences, Cumming School of Medicine, Calgary, AB, Canada; 4 576770 British Columbia Association of Farmers’ Markets, Vancouver, BC, Canada; 5 Future Cities Institute, School of Planning, 8430 University of Waterloo, Waterloo, ON, Canada

**Keywords:** Randomized controlled trial, diet quality, food insecurity, lower-income, farmers’ market, healthy food subsidy, adults, children, parental shielding, dose-response

## Abstract

**Background:**

The British Columbia Farmers’ Market Nutrition Coupon Program (FMNCP) provides lower-income households with coupons to purchase healthy foods at farmers’ markets.

**Aim:**

This pragmatic randomized controlled trial (RCT) will examine the effectiveness and cost-effectiveness of two higher subsidies for healthy food, compared to the usual FMNCP healthy food subsidy, on the diet quality (primary outcome), food insecurity, and other health-related outcomes of 276 parent–child dyads with lower incomes.

**Methods:**

In this single-blind, three-arm, parallel-group pragmatic RCT, 276 parent–child dyads in lower-income households will be randomized to receive the usual FMNCP healthy food subsidy ($27/week) or a subsidy that is two ($54/week) or three times ($81/week) higher. Participants will receive 16 weeks of coupons that can be redeemed for up to 20 weeks. Outcomes will be assessed in one parent (18–64 years) and child (6–17 years) from each household pre-, mid-, and post-intervention. Dietary intake will be collected via two 24-h dietary recalls at each time point to calculate diet quality using the Healthy Eating Food Index-2019. Food insecurity and other health-related outcomes will be assessed using validated self-reported instruments. Mixed effects regression will assess the effects of the interventions, relative to the usual subsidy, on outcomes in parents and children. A full incremental analysis will quantify the cost-effectiveness ratios of the healthy food subsidies.

**Summary:**

This dose-response RCT will show how the diet quality, food insecurity, and other health-related outcomes of parents and children vary according to subsidy amount.

**Trial registration:**

The trial is registered at www.clinicaltrials.gov (NCT06992947).

## Introduction

The high and continually escalating costs of food and other necessities in the face of inadequate wages and social supports prevent many lower-income households from accessing sufficient nutritious food (PROOF, 2026). Household food insecurity refers to inadequate or insecure access to food due to financial constraints. Experiences of food insecurity can range from worrying about running out of food (marginal), to compromising diet quality and/or quantity (moderate), to skipping meals or not eating for an entire day (severe). In Canada, nearly one-quarter of individuals live in a food-insecure household, including nearly a third of children (PROOF, 2026). A large body of evidence confirms that children and adults living in food-insecure households have poorer diet quality (Hutchinson and Tarasuk, 2022; Kirkpatrick et al., 2015; Kirkpatrick and Tarasuk, 2008) and are at higher risk of a range of adverse physical (Men et al., 2020a, 2020b; Te Vazquez et al., 2021) and mental health conditions (Jessiman-Perreault and McIntyre, 2017; Melchior et al., 2012; Men et al., 2021) than their food secure counterparts.

The inadequacies of food banks in reducing food insecurity (Holmes et al., 2018; Middleton et al., 2018; Oldroyd et al., 2022) have led to the development of alternative healthy food subsidy programs that allow recipients to select and purchase their own food in a dignified and autonomy-enhancing manner. Healthy food subsidies offered at farmers’ markets may be particularly beneficial, as shopping in farmers’ markets promotes intake of fruits and vegetables in adults and children with low incomes (Gans et al., 2018; Gorham et al., 2015; Kelley et al., 2022). This may be because the produce sold at farmers’ markets is fresher and tastier, and there are fewer competing ultra-processed foods on offer (Fitting et al., 2022; Freedman et al., 2016; Larsen and Gilliland, 2009). Farmers’ markets also act as community hubs; they enhance social connectedness by facilitating interactions among community members, which may improve mental well-being (Caron-Roy et al., 2022b; Freedman et al., 2016). Purchases made at farmers’ markets also support expansion of local food systems and economies, which provides environmental benefits (British Columbia Association of Farmers’ Markets, 2012; Freedman et al., 2014; Lindsay et al., 2013; Payne et al., 2013). Thus, offering healthy food subsidies at farmers’ markets may generate mutually reinforcing dietary, health, social, economic, and environmental benefits.

The British Columbia (BC) Farmers’ Market Nutrition Coupon Program (FMNCP) is the largest government-funded healthy food subsidy program in Canada. The program provides >10,000 lower-income households in BC with 16 weeks of coupons valued at CAD$27/week to purchase healthy foods at farmers’ markets, and optional nutrition skill-building activities between June and December each year (BC Association of Farmers’ Markets, 2026). In a 2019 randomized controlled trial (RCT; *n* = 285 adults), we found that the FMNCP reduced household food insecurity by 79% (Aktary et al., 2024). However, we were surprised to find that the FMNCP had no impact on the diet quality of adults (Aktary et al., 2023), despite the fact that participants in our concurrent qualitative study reported that the FMNCP improved their households’ diet quality, food security and well-being (Caron-Roy et al., 2022a, 2022b).

Parental shielding occurs when parents protect their children from experiencing the negative effects of food insecurity and takes place in >40% of food-insecure households with children in Canada (Ovenell et al., 2022). Mothers, in particular, compromise their own intake to feed their children (Bell et al., 2022; Hanson and Connor, 2014; McIntyre et al., 2003; Nord, 2013). Based on these data, we hypothesize that parents in our 2019 RCT used their subsidies to purchase additional nutritious foods for their households, thereby reducing household food insecurity. However, because the subsidies were small, they continued to sacrifice their own intake to feed their children, and thus their own diet quality did not improve. This hypothesis is supported by evidence that 43% of parents in our 2019 RCT shielded their children from food insecurity, and by our qualitative findings whereby parents spoke of the benefits of the FMNCP for their children but also recommended providing higher subsidies to better meet their household's food needs (Caron-Roy et al., 2022a, 2022b). Similarly, one supermarket-based RCT in France found that the diet quality of children, but not their parents, improved while receiving a small food subsidy (Buscail et al., 2018). Nevertheless, although evidence suggests that parents preferentially spend food subsidies on their children, we have previously shown that the diet quality of adults in Canada improves with increasing income (Olstad et al., 2021). This indicates that at some point parents must also use additional income to purchase healthier food for themselves, and not just for their children. Taken together, this body of evidence suggests that the diet quality and food security of both parents and children may improve when higher subsidies that satisfy a greater proportion of household food needs are provided. However, the thresholds above which this occurs are unknown, as we are not aware of any published dose-response RCTs in relation to healthy food subsidies among parent–child dyads.

This RCT will examine the effectiveness and cost-effectiveness of two higher subsidies for healthy food, compared to the current usual FMNCP healthy food subsidy, on the diet quality (primary outcome), other dietary outcomes, food insecurity, and self-reported health-related outcomes of 276 parent–child dyads with lower incomes. We hypothesize that: (1) diet quality, other dietary outcomes, and self-reported health-related outcomes will improve in children with increasing subsidy amount; (2) food insecurity (household, parent, and child) will decline with increasing subsidy amount; and (3) diet quality, other dietary outcomes, and self-reported health-related outcomes will improve in parents in the two highest subsidy groups. Figure 1 depicts the pathways through which we posit that changes in study outcomes will be realized.

**Figure 1.**
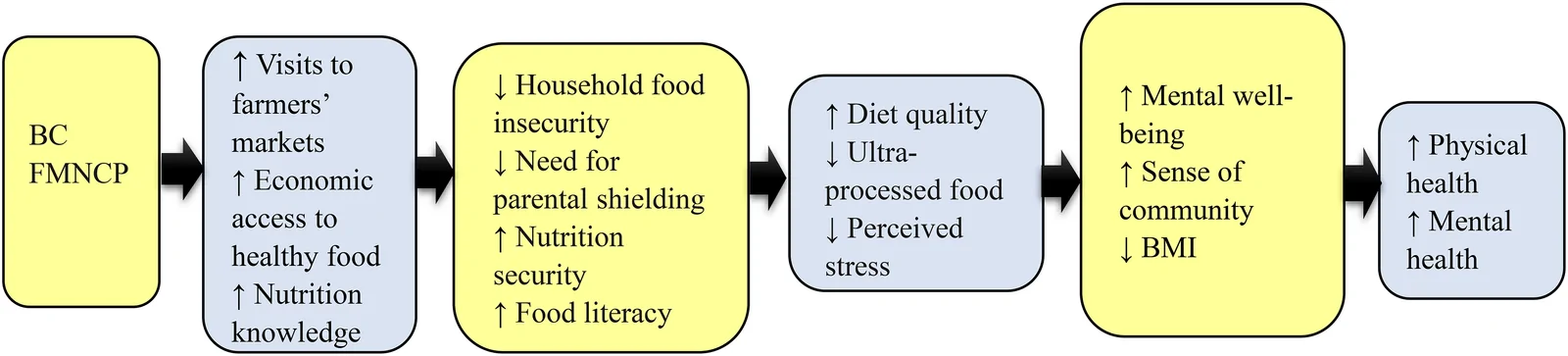
Logic model.

## Methods

### Study design

This RCT was co-designed with the BC Association of Farmers’ Markets, who have been managing and delivering the FMNCP since 2007. This will be a single-blind, three-arm, parallel-group, pragmatic RCT in which 276 parent–child dyads in lower-income households will be randomized to a usual subsidy control group or a group that receives a subsidy that is two or three times higher than the current usual subsidy. The study will be conducted during the 2025 FMNCP season (June–December). As a pragmatic RCT, the intervention will be embedded within the existing FMNCP and will adhere to its standard procedures, including its household-based subsidy model and coupon distribution and redemption timelines. This pragmatic design will maximize the policy relevance of our findings.

This research was approved by the University of Calgary Conjoint Health Research Ethics Board (REB24-1774). Researchers will obtain voluntary, written informed consent from parents for themselves and their child to participate, and children will provide assent. This protocol adheres to SPIRIT reporting guidelines (Supplemental File 1).

### Participants and recruitment

Local community partner organizations that support the health and food needs of target populations (e.g. local food banks) are responsible for enrolling households into the FMNCP and delivering coupons. Community partners in all 96 FMNCP communities will be invited to recruit participants for the RCT. Potentially eligible households will complete an online pre-screening survey. Researchers will contact households that pass the pre-screening survey to confirm final eligibility.

Households will be eligible to participate in the trial if they meet existing FMNCP community-specific annual household income eligibility cut-offs that account for the local cost of living, have two to five household members, have a parent or caregiver 18–64 years who is not pregnant/breastfeeding/trying to conceive and a child 6–17 years, have access to the internet and a camera, are the only dyad in their household participating, are not participating in other trials that might interfere, do not reside in a facility that provides meals (e.g. shelter, long-term care) or where they cannot prepare meals (e.g. hotel), and do not plan to be outside of their community for >2 consecutive weeks, move to a non-FMNCP community, or significantly change their household income or composition during the trial. Parent–child dyads must reside together ≥5 days/week and be able to communicate in English or have someone to translate. If there are multiple eligible individuals, the parent who is the primary food shopper and the youngest child will be enrolled. Children 6–12 years will be preferentially enrolled as they are better able to self-report outcomes than younger children and are more likely to be shielded from household food insecurity than older youth (Hutchinson and Tarasuk, 2022).

### Sample size calculation

Our analyses are powered to detect a between-group difference of three points on the 80-point Healthy Eating Food Index-2019 (HEFI-2019) (Brassard et al., 2022a), which corresponds to an effect size of 0.3. Such a difference can be achieved by, for instance, reducing intake of free sugars or saturated fats from 13% to 10% of energy or reducing sodium by 400 mg/day—differences that are feasible to achieve via each incremental subsidy amount that we are providing and are associated with improved health (Clifton and Keogh, 2017; Dall et al., 2009). Using the flexible power analysis of longitudinal cluster RCT approach that accounts for within-household correlation and within-individual autocorrelation between baseline and the end of the intervention (Wang et al., 2021), 276 parent–child dyads (*n* = 92 dyads per group) are required to detect at least a three-point between-group difference in HEFI-2019 scores, assuming a standard deviation of the between-group difference in HEFI-2019 scores of 9.2 (Brassard et al., 2022a), 90% statistical power, intra-class correlation of 0.15 (Beydoun and Wang, 2009; Bogl et al., 2017; Wang et al., 2011), within-cluster correlation of 0.70 and 20% attrition.

### Randomization

Following baseline data collection, households will be randomized 1:1:1 to one of three groups (*n* = 92 households/group) using a computer-generated, concealed permuted block randomization scheme with a block size of three designed by an independent statistician. Randomization will be stratified by expected coupon redemption period as farmers’ markets in different communities open and close at different times (<16 weeks, 16–19 weeks, 20–22 weeks, and 23–26 weeks), region (rural, small/medium urban, and large urban), and household size (2–3 and 4–5). Allocation assignment will be operationalised via Research Electronic Data Capture (REDCap), and group assignments will be communicated by email to participants and the FMNCP to maintain researcher blinding. Allocation concealment will be ensured by storing the randomization scheme separately from the participant database in a manner only accessible to the statistician.

### Intervention

Households will be randomized to receive one of three subsidies to purchase healthy foods at farmers’ markets for up to 20 weeks: (1) SUBSIDY-USUAL: $27/week; (2) SUBSIDY-2: $54/week; and (3) SUBSIDY-3: $81/week (Figure 2). In keeping with the existing FMNCP, participants will be eligible, but not required, to participate in nutrition skill-building activities such as cooking and gardening classes.

**Figure 2.**
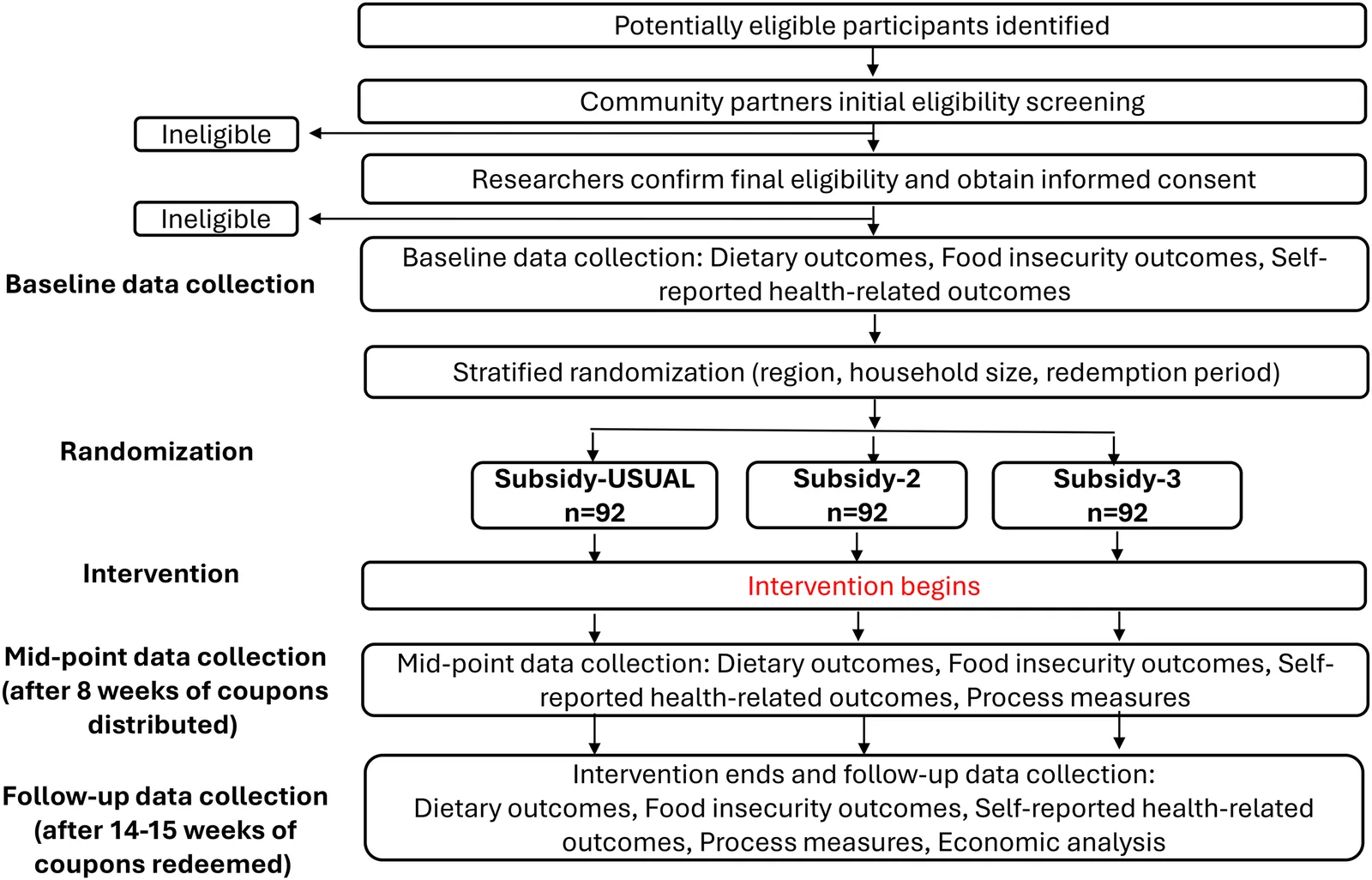
Study flow diagram.

SUBSIDY-USUAL will receive the current usual FMNCP subsidy of $27/household/week. This is similar to the usual subsidy of $21/household/week that was tested in our 2019 RCT after adjusting for food inflation (Statistics Canada, 2026). The increments between subsidy levels are large enough to meaningfully improve food purchasing power, yet small enough to allow us to establish a minimum effective dose. Each incremental dose is estimated to allow households to purchase an additional ∼9% of the contents of a nutritious food basket in BC in 2025 (BC Centre for Disease Control, 2022). This incremental purchasing power is significant given that the FMNCP is meant to supplement household food budgets and not to cover all household food expenses.

The intervention will be delivered as part of the existing FMNCP whereby community partners will identify and enroll households into the program, distribute coupons and provide optional nutrition skill-building activities. Coupons will be provided in $3 denominations and can be used to purchase a variety of healthy foods, including vegetables, fruits, dairy, meat, poultry, fish, eggs, nuts, vegetable and fruit plants, honey, and herbs. Trained vendors at farmers’ markets will redeem coupons. Households will receive 16 weeks of paper-based coupons beginning in June or July 2025, depending on their enrollment date. Coupon distribution schedules vary by community partner (e.g. weekly, bi-weekly) and the duration of the farmers’ market season in each community. Coupons do not expire and can be redeemed for up to 20 weeks until 21 December 2025, although some farmers’ markets close earlier in the season and thus redemption periods vary across communities.

### Data collection

Data will be collected pre-, mid- (i.e. after 8 weeks of coupons have been distributed) and post-intervention (i.e. after participants have redeemed 14–15 weeks of coupons) to provide a comprehensive perspective of the impact of the FMNCP throughout the farmers’ market season and whether it varies over time. The timing of mid-intervention data collection will be based on the timing of coupon receipt rather than redemption, as participants can theoretically redeem all of their coupons at once or in a small number of visits. This will help to ensure data are collected at three relatively evenly spaced intervals, while individualizing data collection based on each household’s unique program duration. Participants will have access to a dedicated study help line and email where they can report any concerns with the research and receive support with data collection.

The primary outcome is overall diet quality post-intervention in parents and children (Table 1). Secondary outcomes (in households, parents, and/or children) at mid- and post-intervention include: (1) other dietary outcomes: overall diet quality (mid-intervention only), diet quality components, food purchasing, intake of ultra-processed foods, and total energy intake; (2) food insecurity outcomes: food insecurity status and severity, nutrition security, and parental shielding; and (3) self-reported health-related outcomes: mental well-being, sense of community, perceived stress, perceived health, food literacy, and body mass index (BMI). Process measures will encompass coupon receipt and redemption, and attendance at nutrition skill-building activities to understand the dose of intervention received and food purchasing patterns. We will also conduct an economic analysis to calculate the incremental cost-effectiveness ratios of the healthy food subsidies.

**Table 1.** Measurement tools and procedures.

Construct	Instrument, data source
Primary outcome: Assessed pre-, mid-, and post-intervention	
*Overall diet quality: Reported separately for the parent and child*	
Overall diet quality post-intervention	Two 24-h dietary recalls to report dietary intake and supplements taken from midnight to midnight the previous day via the online Automated Self-Administered 24-h Dietary Recall for Canada 2018 (ASA24 Canada, 2020; Kirkpatrick et al., 2014; Subar et al., 2012); values used to calculate Healthy Eating Food Index-2019 scores from 0 to 80 (Brassard et al., 2022a)
Secondary outcomes: Assessed pre-, mid-, and post-intervention	
*Other dietary outcomes: Reported separately for the parent and child*	
Overall diet quality mid-intervention	As above, two 24-h dietary recalls used to calculate Healthy Eating Food Index-2019 scores from 0 to 80 (Brassard et al., 2022a)
Ten diet quality components	As above, two 24-h dietary recalls used to calculate Healthy Eating Food Index-2019 component scores from 0 to 5 or from 0 to 10 (Brassard et al., 2022a)
% energy consumed from ultra-processed foods	As above, two 24-h dietary recalls used to calculate % of energy from ultra-processed foods using Nova classification (Monteiro et al., 2019; Steele et al., 2023)
Total energy intake	As above, two 24-h dietary recalls used to calculate energy intake
% of coupons (based on dollar value) used to purchase foods with higher and lower quality	Participants will take photos of all foods purchased with coupons using a study-provided Web App; foods will be classified as: (1) minimally processed, culinary ingredients, processed, and ultra-processed using Nova (Monteiro et al., 2019; Steele et al., 2023); (2) very poor, poor, fair, good, and excellent choice using the Canadian Food Scoring System (Lee et al., 2024); and (3) serve frequently and serve infrequently using the BC School Food Toolkit (Healthy Schools BC, 2023)
*Food insecurity outcomes: Reported by parents at the household level based on the past 30 days*	
Food insecurity status	Household Food Security Survey Module (food secure and food insecure) (Health Canada, 2012, 2020)
Food insecurity severity	Household Food Security Survey Module (food secure, marginal, moderate, and severe food insecurity) (Health Canada, 2012, 2020)
Nutrition security status	Nutrition Security Scale (low nutrition security and high nutrition security) (Calloway et al., 2022; Gretchen Swanson Center for Nutrition, 2023)
Parental shielding	Household Food Security Survey Module (shielded and not shielded; shielding exists when there are affirmative responses on the adult but not the child scale) (Health Canada, 2012, 2020)
*Self-reported health-related outcomes: Reported separately for the parent and child*	
Mental well-being	Parent: Warwick-Edinburgh Mental Well-Being Scale (Castellvi et al., 2014; Clarke et al., 2011; Tennant et al., 2007; Warwick Medical School, 2016); Child: Stirling Children's Wellbeing Scale (Liddle and Carter, 2013, 2015)
Sense of community	Parent: Brief Sense of Community Scale (Peterson et al., 2008); Child: not assessed
Perceived stress	Parent: Perceived Stress Scale-10 (Cohen et al., 1983; Cohen and Williamson, 1988; Lee, 2012); Child: Perceived Stress Scale for Children (White, 2014)
Self-rated health	Parent and Child: Single-item self-rated health question (Jylha, 2009; Statistics Canada, 2023a, 2023b)
Food literacy	Parent: Canadian Food Literacy Measure (Martin et al., 2024); Child: not assessed
BMI	Parent: Self-reported height and weight, BMI is weight in kg divided by height in metres squared; Child: Self-reported height and weight used to calculate BMI *z*-score (World Health Organization, 2007)
Process measures: Assessed throughout the intervention	
*Process measures: Reported at the household level*	
Coupon receipt	Community partner and researcher records
Coupon redemption	Farmers’ market vendors retain coupons (coupons have serial numbers linked to participant ID); participants take photos of foods purchased with coupons using a study-provided Web App
Nutrition skill-building	Community partner records
*Sociodemographic and health-related variables: Assessed pre-, mid-, and post-intervention; reported separately for the parent and child or at the household level*	
We will use validated questions from the Canadian Community Health Survey (Statistics Canada, 2022) except where indicated: date of birth, sex at birth, gender identity (Cameron and Stinson, 2019), ethnicity, immigration status, years lived in Canada, smoking, physical activity level (Milton et al., 2011; Scott et al., 2015), number of children and adults in the home, annual household income, perceived income adequacy (Gildner et al., 2019; Hagenaars and de Vos, 1988; Litwin and Sapir, 2009), highest household educational attainment, parental marital status, parental employment status, participation in other income/food support programs, prior participation in the FMNCP, housing status, community of residence, and name of the farmer's market where participants plan to redeem their coupons.	

BC: British Columbia; BMI: body mass index; FMNCP: Farmers’ Market Nutrition Coupon Program.

At all three time points, parents and children will jointly complete an online questionnaire in REDCap to report sociodemographic variables, food insecurity, and other health-related outcomes using valid and reliable tools that are sensitive to change (Table 1). The final questionnaire was reviewed by all authors for face and content validity and was pre-tested with two parents and one child.

At baseline, parents and children will report dietary intake and supplements taken from midnight to midnight the previous day via the online Automated Self-Administered 24-h Dietary Recall for Canada 2018 (ASA24-Canada-2018) (Table 1) (ASA24 Canada, 2020; Kirkpatrick et al., 2014; Subar et al., 2012). Twenty-four-hour recalls are the recommended method of dietary assessment for interventions, as they have less systematic bias than other self-report instruments (Kirkpatrick et al., 2022). The ASA24-Canada-2018 corresponds well with interviewer-administered dietary recalls and with true intakes in adults (Kirkpatrick et al., 2014; Park et al., 2018). However, some children have difficulty reporting their intake (Baranowski et al., 2012; Raffoul et al., 2019; Sharpe et al., 2021). Data indicate that parents can accurately report their children's intake using the ASA24-Canada-2018 (Sharpe et al., 2021; Wallace et al., 2018). Therefore, parents will assist children to report their intake. Participants will complete two dietary recalls at each time point after receiving a phone/email prompt, each separated by 3–6 days. Collecting two recalls at each time point will provide a more precise estimate of how the FMNCP affects diet quality—even among those who do not redeem coupons every week. Prior to the first recall at mid- and post-intervention, participants will be asked to report the total value of coupons they redeemed in the previous 7 days. Participants who require assistance or who do not have internet access can complete their recalls with researchers by phone. Researchers will also review completed questionnaires and dietary intake data within 24 h of receipt and will clarify implausible or incomplete responses with participants.

Dietary intake data from both recalls at each time point will be averaged to derive HEFI-2019 total and component scores to provide a valid assessment of diet quality consistent with the 2019 Canada Food Guide (Brassard et al., 2022a, 2022b). Dietary intake data will also be used to calculate intake of ultra-processed foods (% energy) using the Nova classification (Monteiro et al., 2019; Steele et al., 2023) and energy intake. The proportion of coupons (based on dollar value) spent on foods with higher and lower nutritional quality will be assessed by asking participants to take photos of all items purchased with their coupons using a study-provided Web App.

Process measures will be assessed through self-reported data from parents, including photos of foods purchased with coupons, objective data on coupon redemption obtained from vendors at farmers’ markets (coupons have serial numbers linked to participant ID), and community partner and researcher records of coupon distribution and skill-building activities (Table 1). To support retention, households will receive a $100 gift card for providing data at each time point. To avoid interfering with study outcomes, all gift cards will be distributed after the RCT is complete. Households that drop out will be asked to provide a reason to assess if dropout was related to the intervention or study outcomes.

### Blinding

Participants cannot be blinded to group assignment; however, to minimize biases, we will not discuss details of our specific interventions (e.g. participants will know they will receive at least the usual coupon amount but not the exact value of the two higher subsidy levels), objectives, or hypotheses. To ensure community partner blinding and minimize group contamination, FMNCP staff will place each household's coupons in a sealed envelope for distribution by community partners. Community partners will be instructed not to discuss group allocation with participants. Vendors who redeem coupons at farmers’ markets will also be blinded to group allocation. Researchers who collect and analyze the data will not be involved in delivering the intervention and thus will remain blinded.

### Data analysis

There will not be a Data Monitoring Committee or interim analyses as the intervention is minimal risk and unlikely to have significant adverse impacts. A detailed Statistical Analysis Plan will be posted to the clinicaltrials.gov registry prior to conducting the analyses.

Descriptive statistics will be calculated for all variables and compared to those of the full FMNCP to understand the generalizability of our findings. Analyses will be intention-to-treat, in which participants will be analyzed within their assigned groups regardless of adherence or dropout. For the primary analysis, a linear mixed effects regression model will assess between-group differences in HEFI-2019 scores post-intervention among parents and children. Models will include intervention group, time, and an intervention group-by-time interaction as fixed effects. Repeated measures within participants (level 1) will be modelled with a random intercept and random slope for time; clustering of participants within households (level 2) will be modelled with a random intercept. Models will be adjusted for randomization stratification variables, day of dietary recalls, and baseline covariates, including age, gender, race/ethnicity, and Indigenous status. For secondary outcomes, generalized linear mixed models with appropriate distribution and link functions will examine the effects of intervention group on continuous, binary, and ordinal outcomes adjusted for outcome-specific covariates. If significant differences are found, linear contrasts will be used to conduct pairwise comparisons of each arm to SUBSIDY-USUAL. In addition, given that the current RCT does not have a no-intervention control group, we will also examine whether diet quality, energy intake, household food insecurity, mental well-being, and sense of community among adults in the current RCT differ from those among adults in the control group in our 2019 RCT. All analyses will be evaluated at the α = 0.05 significance level.

Exploratory subgroup analyses will examine whether the effects of the intervention differed by gender, child age group, household size, household food insecurity, and actual coupon redemption period. If effect modification is found, we will report stratum-specific estimates.

Multiple imputation and other missing data methods will be used to determine the robustness of our findings to assumptions about missing data. In sensitivity analyses, we will run separate models for parents and children, adjust for the average value of coupons redeemed in the 7 days preceding dietary recalls, exclude dietary recalls if participants redeemed an average of <$27 in coupons in the 7 days prior to the first dietary recall, use the Healthy Eating Index-2020 as an alternate measure of diet quality, adjust for an indicator of dietary misreporting (i.e. the ratio of total energy intake to total energy expenditure (Bajunaid et al., 2025; Huang et al., 2005)), restrict the analyses to participants whose actual coupon redemption period was close to the intended redemption period, use % gram weight as an alternate measure of ultra-processed food intake, and conduct per-protocol and as-treated analyses.

### Cost-effectiveness analysis

We will examine the incremental cost-effectiveness of the usual and two higher subsidies for healthy food using the cost for a three-point increase in HEFI-2019 scores and the cost per 1% decline in household food insecurity. These outcomes will be directly quantified in the RCT, reducing the uncertainty and potential bias that could occur if we were to extrapolate to longer-term health or mortality outcomes. Canada's Drug Agency Health Economic Evaluation Methods Guide (Canada's Drug Agency, 2017) will be followed using a healthcare payer perspective. We will include all administrative costs and payments to participants. Results will be probabilistic, accounting for the uncertainty in the trial. A second fully incremental analysis among adults will compare the cost-effectiveness of the three healthy food subsidies in the current trial to the no-intervention control group in our 2019 RCT. Sensitivity analyses will examine the effect of parameter and scenario uncertainty on the outcomes and cost-effectiveness.

## Summary

Effective and sustainable policies are needed to reduce the tremendous burden that poor diet quality and food insecurity impose on the health of Canadians and associated high healthcare costs borne by all (Fitzpatrick et al., 2015; Tarasuk et al., 2015). Our 2019 RCT left unanswered questions regarding the causal impacts of healthy food subsidies at farmers’ markets—and the FMNCP in particular—because it examined the impact of a single, small usual subsidy among adults alone. The current dose-response RCT is uniquely capable of answering these questions as it will show how the diet quality, food insecurity, and other health-related outcomes of parents and children vary according to subsidy amount. Some outcomes, such as food insecurity and self-reported health-related outcomes, may improve with smaller subsidies as greater access to even small amounts of food can ameliorate them (e.g. by reducing financial stress). Other outcomes, such as diet quality and BMI, may only improve if larger subsidies are provided, as they cannot improve unless individuals regularly consume adequate nutritious foods. Moreover, because parents attempt to shield their children from food insecurity, smaller subsidies may only affect outcomes among children, whereas larger subsidies may be required to improve outcomes among parents. We have already shown that the usual FMNCP subsidy reduces household food insecurity (Aktary et al., 2024)—the key mediator of the beneficial dietary and health effects of healthy food subsidies (Bell et al., 2022; Seligman and Schillinger, 2010; Te Vazquez et al., 2021). Each incrementally higher subsidy dose in the current trial should therefore further reduce food insecurity and thereby improve diet quality and other outcomes among parents and/or children. The subsidy thresholds above which these changes occur are likely to differ by outcome and between parents and children. The advantage of our dose-response design is that it is uniquely capable of demonstrating how variations in subsidy amounts affect distinct outcomes among parents and children separately. Evidence of dose-response can also provide stronger evidence of causality.

This study will be the first to show whether a scalable, government-funded, population-level healthy food subsidy can improve the diet quality, food security, and other health-related outcomes of parents and children with lower incomes, and the dose needed to realize such benefits. To improve external validity, we will use a pragmatic design in which we will recruit participants who are already participating in or are eligible for the regular FMNCP, deliver the intervention using existing FMNCP resources, and adhere to all standard FMNCP procedures. Study findings will be vital to assist policymakers in calibrating food subsidy levels to their policy aims. For instance, a food subsidy that aims to improve food security among children can likely be smaller than one which aims to improve the diet quality and food security of all household members—as the FMNCP does. We may also find a ceiling effect whereby outcomes do not continue to improve above a certain subsidy amount—indicating that higher subsidies may not be needed. Due to necessary constraints on public health spending, policymakers may wish to use evidence from a dose-response study such as ours to understand the minimum subsidy amount that can contribute to their policy aims. As there is no standard willingness-to-pay for improvements in diet quality and food security, our cost-effectiveness analysis may also set a standard to which other public health interventions can be compared to understand the most cost-effective ways to improve diet quality and food security. To increase the generalizability of our results, we will present them in terms of the proportion of a nutritious food basket that each subsidy level allowed households to purchase, and how outcomes varied across the subsidy levels. Thus, our findings will yield generalizable principles to inform the design of healthy food subsidies more broadly.

Although we are using the recommended dietary assessment method for interventions (i.e. 24-h dietary recalls), social desirability bias is inherent within all self-reports. Weight and height will also be self-reported. However, the tendency to misreport intake, weight, and height should be balanced between groups owing to randomization. In sensitivity analyses, we will also adjust dietary intake for an indicator of misreporting. Some participants may feel that the restriction to healthy foods is paternalistic. However, restricting food subsidies to the purchase of healthy foods is an evidence-based practice essential to avoid the potential unintended negative consequence of increasing purchase of unhealthy foods. Moreover, as a pragmatic trial, we must adhere to the current FMNCP funding model.

It is useful to study the impact of government programs to understand their effectiveness and if changes and innovations are required to improve their effectiveness, particularly before scaling them up. A key advantage of the current RCT is that we are not studying a hypothetical program designed by researchers, but one that has proven to be both practically and politically viable since 2007. Our dose-response design will allow us to discern how variations in subsidy amounts affect distinct outcomes among parents and children separately, enabling novel insights that will assist policymakers to calibrate food subsidy levels to their policy aims.

## Supplemental Material

sj-doc-1-nah-10.1177_02601060261473062 - Supplemental material for Does the British Columbia Farmers’ Market Nutrition Coupon Program improve the diet quality and food security of parents and children with lower incomes? Protocol for a dose-response, pragmatic randomized controlled trial Supplemental material, sj-doc-1-nah-10.1177_02601060261473062 for Does the British Columbia Farmers’ Market Nutrition Coupon Program improve the diet quality and food security of parents and children with lower incomes? Protocol for a dose-response, pragmatic randomized controlled trial by Dana Lee Olstad, David JT Campbell, Tolulope T Sajobi, Heather O’Hara, Peter Leblanc, Gavin R McCormack, Leticia Radin-Pereira, Yun Yun Lee, Michelle L Aktary, Leia M Minaker and Eldon Spackman in Nutrition and Health

## Data Availability

Data will be available from the authors upon reasonable request.
